# An unexplored equity factor: differential beliefs and attitudes toward contingency management by providers’ ethnicity

**DOI:** 10.1186/s12913-023-09878-7

**Published:** 2023-08-23

**Authors:** Oladunni Oluwoye, Douglas L. Weeks, Michael G. McDonell

**Affiliations:** Elson S. Floyd College of Medicine, Washington State University, Spokane, WA 99202 USA

**Keywords:** Contingency management, Ethnicity, Implementation, Providers, Substance use treatment

## Abstract

**Background:**

Although considered one of the most effective interventions for substance use disorders (SUD), the widespread implementation of contingency management (CM) has remained limited. In more recent years there has been surge in the implementation of CM to address increasing rates of substance use. Prior studies at the provider-level have explored beliefs about CM among SUD treatment providers and have tailored implementation strategies based on identified barriers and training needs, to promote implementation of CM. However, there have been no implementation strategies that have actively sought to identify or address potential differences in the beliefs about CM that could be influenced by the cultural background (e.g., ethnicity) of treatment providers. To address this knowledge gap, we examined beliefs about CM among a sample of inpatient and outpatient SUD treatment providers.

**Methods:**

A cross-sectional survey of SUD treatment providers was completed by 143 respondents. The survey asked respondents about their attitudes toward CM using the Contingency Management Beliefs Questionnaire (CMBQ). Linear mixed models examined the effect of ethnicity (non-Hispanic White and Hispanic) on CMBQ subscale (general barriers, training-related barriers, CM positive-statements) scores.

**Results:**

Fifty-nine percent of respondents to the CMBQ self-identified as non-Hispanic White and 41% as Hispanic. Findings revealed that treatment providers who identified as Hispanic had significantly higher scores on the general barriers (p < .001) and training-related barriers (p = .020) subscales compared to the non-Hispanic White treatment providers. Post-hoc analyses identified differences in the endorsement of specific individual scale items on the general barriers (e.g., CM interventions create extra work for me) and training-related (e.g., I want more training before implementing CM) subscales.

**Conclusions:**

Dissemination and implementation strategies for CM need to consider equity-related factors at the provider-level that may be associated with the adoption and uptake of CM.

**Supplementary Information:**

The online version contains supplementary material available at 10.1186/s12913-023-09878-7.

## Introduction

Contingency management (CM) is an evidence-based intervention for substance use disorders (SUD) that provides positive reinforcement (e.g., prizes, vouchers, gift cards) for targeted behaviors such as abstinence or the reduction of substance use [1]. Multiple studies have demonstrated the effectiveness of CM for SUD in community-based settings [2–4]. CM has also been culturally adapted to meet the treatment needs of specific ethnoracial groups (e.g., American Indian/Alaska Native, Hispanic) and has been effective in reducing substance use among ethnoracial minorities [5–7]. More recently, there has been an increasing demand for CM, as a highly effective evidence-based intervention to address the rising rates of stimulant and opioid use throughout the U.S. [8, 9]. Although previous research has demonstrated the effectiveness of CM in multiple practice settings, the widespread implementation of CM has been slow [10].

Pre-implementation is characterized as the work needed to understand potential barriers and facilitators to implementing evidence-based interventions among providers which often includes the knowledge of and beliefs about an intervention [11–14]. Treatment providers’ beliefs and attitudes towards CM have been well documented and are an important individual-level factor that can impede or facilitate implementation [15]. Several studies among a general sample of SUD treatment providers, have found that negative attitudes towards the philosophical underpinnings, operational cost, lack of training, and limited knowledge about CM were barriers [16–18]. Further, certain provider characteristics, such as educational attainment and years of experience, have also been shown to influence beliefs about CM [18, 19]. For instance, Kirby and colleagues [18] reported that providers with advanced degrees (e.g., masters, doctoral) had more positive beliefs about CM compared to providers with bachelor-level degrees. Based on what is known about providers’ beliefs and attitudes towards CM, implementation strategies have been developed to include pragmatic training components to target many of these factors, such as lack of training (e.g., CM delivery) and limited knowledge (e.g., theoretical underpinnings of CM) [15]. However, many of these earlier studies examining beliefs and attitudes toward CM and assessing implementation strategies have not considered equity-related factors such as the race and ethnicity of providers.

Interestingly, only one CM focused study has been conducted and examined ethnoracial differences in the adoption of CM. Helseth and colleagues [20] reported lower CM adoption rates among SUD providers who identified as an ethnoracial minority relative to non-Hispanic White providers. While there has been an indication that ethnoracial identity may have some impact on the implementation of CM, it unknown whether this specific provider-level characteristic affects beliefs and attitudes toward CM which is important to understanding the successfulness of implementation.

In other behavioral health fields, there have also been few studies that have found an association between the race and/or ethnicity of providers and attitudes towards the general use of evidence-based interventions. For instance, Aarons and colleagues’ findings suggest that Black and Hispanic providers report more negative attitudes toward evidence-based interventions compared to White providers [21, 22]. In contrast to these studies, one study found that Hispanic providers reported more positive attitudes toward evidence-based interventions in community mental health settings [23]. To our knowledge no studies have explored the relationship between providers’ race and/or ethnicity and their beliefs about CM. Thus, the purpose of this study was to conduct a survey to characterize beliefs about CM among Hispanic and non-Hispanic White SUD treatment providers in community-based settings.

## Methods

### Participants and procedures

Potential participants were eligible if they were 18 years of age and older, self-identified as currently or previously employed as a provider in an addiction treatment clinic, and reported English language fluency. Potential participants did not need to have prior experience with CM to complete the online survey. Between January 2019 and June 2019, a total of 203 potential participants responded to a targeted email disseminated to professional groups focused on SUD and addiction (i.e., Addiction Drug and Alcohol Institute), who were asked to also share it with other networks. Of those who initially responded, 162 respondents met inclusion criteria, with 143 providing responses on the Contingency Management Beliefs Questionnaire (CMBQ), which is described in detail below, for a participation rate of 88.3% [24].

To facilitate survey completion, a Research Electronic Data Capture (REDCap)-based electronic survey was used to capture responses from eligible participants and the survey link was embedded in the email distributed to listservs. The result was an “opt-in” nonprobability sample based on participants recruited through the professional group listserv sampling frame. This study was considered exempt by the university’s Institutional Review Board and participants were asked to read and download informed consent forms and indicate whether they would like to continue the survey. At the end of the survey, participants were asked to provide their email address to receive a $15 e-gift card for survey completion. The survey was closed after six months of recruitment and all data were maintained in a secure electronic database.

### Measures

#### Contingency management beliefs questionnaire

The 32-item CMBQ was used to assess the level of influence each item has on providers’ decision to adopt the use of CM on a five-point Likert scale (0 = no influence at all; 1 = very little influence; 2 = some influence; 3 = strong influence; 4 = very strong influence). Exploratory and confirmatory factor analysis of CMBQ item scores suggests a stable and reliable three factor structure forming three subscales totaling 32 items: General Barriers (17 items), Training-related Barriers (4 items), and CM-supportive Statements (11 items) [25]. The general barriers subscale included items related to time and cost demands of implementing CM, as well as clinical concerns. Training-related barriers pertained to lack of training opportunities and qualified supervision, as well as concerns about organizational support. The CM-supportive statements related to the perceived benefits of CM.

#### Demographics

Data were also collected on key demographic characteristics, such as age, gender (male, female, transgender, or nonbinary of which respondents only selected male or female thus creating a binary variable), race (White, Black/African American, Asian, American Indian/Alaska Native, Native Hawaiian or Pacific Islander, Other), ethnicity (non-Hispanic or Hispanic), licensed mental health counselor (LMHC) credential status (yes/no), and graduate degree status (yes/no).

### Data analysis

Among respondents who met study inclusion criteria (n = 162), 49% self-identified as non-Hispanic White, 34% as Latinx, 7% as Black, and 10% as another racial minority. Based on the sparse representation of races and ethnicities other than non-Hispanic White and Hispanic ethnicity, we limited our analyses to these two groups in order to avoid error in interpretation from very small subsamples of other races. Demographic comparisons among non-Hispanic White and Hispanic respondents were appropriate to the scale of measurement: age was compared with independent sample t-tests with all other demographic variables analyzed in two-way contingency table analyses using exact tests to assess for significant differences in proportions by ethnicity. For inferential testing, CMBQ subscale scores were converted to the mean scale score by summing scores for all items completed within a scale and dividing by the number of items completed in the scale. Averaging standardized scales (composed of different numbers of items) for comparison and compensated for the limited amount of non-response within a few respondents, as detailed in the Results section.

A linear mixed model was used to examine the effect of ethnicity on mean CMBQ subscale scores. Ethnicity was modeled as a binary fixed factor with the each CMBQ subscale scores, representing levels of a second fixed repeated measures factor - scale. The ethnicity and scale interaction term were also modeled as the primary parameter of interest in order to evaluate whether scores on each subscale differed among non-Hispanic White and Hispanic respondents. The initial fully specified model included all demographic variables that differed significantly by ethnicity in univariate analyses as covariates (i.e., age, gender, credential status, graduate degree status). A second, more parsimonious, model was developed that removed gender and credential status from the model as these were not significant as covariates in the fully specified model (*p* = .941 and *p* = .322, respectively). Graduate degree status and age were maintained as covariates in the second model. Model fit to the data improved in the second model based on a reduction in Akaike’s Information Criterion. Both models employed random intercepts for respondents to account for correlation between CMBQ subscales, restricted maximum likelihood estimation, and modeled covariance structure as a scaled identity matrix; both models converged under these criteria.

A significant ethnicity by CMBQ subscale interaction was followed up with simple main effects tests to determine which scales differed by ethnicity. Post-hoc analyses were conducted on CMBQ subscales that differed by ethnicity using a scale-item by scale-item follow up 2 by 2 (ethnicity by dichotomized item scale score [‘some to very strong influence’ vs. ‘very little to no influence’]) exact tests to explore differences in specific attitudes toward CM among Hispanic and non-Hispanic White respondents. All analyses used two-sided type I error rates of *p* < .05, and were conducted with SPSS, v. 28.0.

## Results

### Participant characteristics

Overall (N = 143), the mean age was 41.0 years, 59% (n = 85) self-identified as non-Hispanic White and 41% (n = 58) as Hispanic. As seen in Table 1, non-Hispanic White respondents were significantly older and had significantly more female providers than participants who identified as Hispanic. A significantly higher proportion of Hispanic respondents were LMHC credentialed, while a significantly higher proportion of non-Hispanic White respondents had graduate degrees. The majority of respondents in both groups worked in addiction or mental health agencies, and most worked in outpatient settings.

**Table 1:** Demographic Characteristics of Respondents

Variable	Overall (N = 143)	Non-Hispanic White (N = 85)	Hispanic (N = 58)	*p*
**Age Mean (SD)**	41.0 (11.6)	43.6 (12.5)	37.1 (8.9)	< 0.001
**Gender %(N)**				< 0.001
Male	54.5% (78)	42.4% (36)	72.4% (42)	
Female	45.5% (65)	57.6% (49)	27.6% (16)	
**Education %(N)**				
Graduate degree	37.1% (53)	50.6% (43)	17.2% (10)	< 0.001
**Agency Type %(N)**				0.293
Inpatient	20.3% (29)	23.5% (20)	15.5% (9)	
Outpatient	79.7% (114)	76.5% (65)	84.5% (49)	
**Credentials %(N)**				
LMHC^1^ Credential	40.6% (58)	18.8% (16)	72.4% (42)	< 0.001
ABPP^2^ Credential	10.5% (15)	9.4% (8)	12.1% (7)	0.782
LICSW^3^ Credential	8.4% (12)	8.2% (7)	8.6% (5)	0.999
**Addiction/Mental Health Agency**	94.4% (135)	90.6% (77)	100% (58)	0.052

^1^ Licensed Mental Health Counselor

^2^ American Board of Professional Psychology

^3^ Licensed Independent Clinical Social Worker

### Differences in CMBQ subscale scores by ethnicity

Linear mixed modeling of CMBQ subscale scores revealed significant main effects by ethnicity (*p* = .001) and the repeated measures scale variable (*p* < .001), after adjustment for age and graduate degree status. These main effects were superseded by a significant ethnicity by CMBQ subscale interaction (*p* < 001). Simple main effects testing revealed significantly higher general barriers scale scores (*p* < 001) and training-related barriers scale scores (*p* = .020) among Hispanic respondents compared to non-Hispanic White respondents. Scores on the CM-Supportive Statement subscale did not differ by ethnicity (*p* = .853). Interaction means derived from linear mixed modeling are displayed in Table 2.

**Table 2:** Group means derived from linear mixed modeling for each Contingency Management Beliefs Questionnaire (CMBQ) scale. 95% confidence intervals are in parentheses

CMBQ Subscales	Non-Hispanic White (n = 85)			Hispanic (n = 58)	
	M	95% CI		M	95% CI
General Barriers	2.78	(2.64, 2.93)		3.41	(3.24, 3.59)
Training-Related Barriers	3.17	(3.03, 3.32)		3.49	(3.27, 3.62)
CM-Supportive Statements	3.35	(3.21, 3.50)		3.33	(1.05, 3.55)

### Differences in individual items on general and training-related barriers subscales

For each of the 17 items in the General Barriers subscale, significantly higher proportions of Hispanic respondents endorsed “some to very strong influence” compared to non-Hispanic White respondents (all *p*-values ≤ 0.036). Items with the greatest differences in endorsement among groups (> 40% difference in group proportions) included external barriers (e.g., clients already abstinent so don’t need CM and clinics prevent urine screening) and internal barriers (e.g., provider finding CM distasteful and clinical experience more important than research evidence). Differences in endorsement of individual subscale items are displayed in Table 3.

**Table 3:** Proportions endorsing “Some to Very Strong Influence” per Contingency Management Beliefs Questionnaire (CMBQ) item within the General Barriers and Training-related Barriers scales. P-values were derived from exact tests comparing responses from the non-Hispanic White and Hispanic respondents

Subscale Items	Non-Hispanic White	Hispanic	*p*
**General Barriers Subscale**			
The research evidence about CM’s effectiveness does not apply to everyday clinic populations.	78.6%	100%	< 0.001
Clients might sell/trade earned items for drugs.	74.1%	89.7%	0.031
A lot of my clients are already abstinent at intake, so they don’t need CM.	43.5%	86.2%	< 0.001
I find CM distasteful because it is basically paying someone to do what they should do already.	45.9%	89.7%	< 0.001
CM is expensive (e.g., cost of prizes, vouchers).	70.6%	94.7%	< 0.001
I am not convinced by the research about CM’s effectiveness with substance abusers.	61.2%	82.8%	0.006
Providing prizes/vouchers undermines the clients’ internal motivation to stay sober.	65.9%	94.8%	< 0.001
I do not have time to administer vouchers/prizes in a therapy session.	54.1%	79.3%	0.002
My clinical experience with recovering substance abusers is more important than any research evidence.	42.4%	82.8%	< 0.001
Clients will view CM as patronizing.	58.8%	93.1%	< 0.001
CM interventions create extra work for me.	57.8%	91.4%	< 0.001
I am worried about what happens once the contingencies are withdrawn.	79.8%	93.1%	0.031
CM might cause arguments among clients (e.g., when some get prizes and other do not).	58.3%	89.7%	< 0.001
I believe it is not right to give rewards for abstinence if clients are not meeting other treatment goals (e.g., group attendance).	58.3%	87.9%	< 0.001
CM doesn’t address the underlying cause of addiction.	65.5%	82.8%	0.035
The community wouldn’t understand (i.e., clinic will look bad for giving rewards to substance abusers).	48.2%	84.5%	< 0.001
Our clinic rules prevent urine screening.	42.9%	89.3%	< 0.001
**Training-Related Barriers Subscale**			
I want more training before implementing CM.	81.2%	94.8%	0.023
I don’t feel qualified or properly trained to administer CM.	73.8%	78.9%	0.551
Currently, no one in my facility has the experience to supervise CM.	73.8%	81.0%	0.419
My agency/supervisors/administrators do not support CM (e.g., do not provide training, resources).	67.1%	87.9%	0.005

On the Training-Related Barriers subscale, significantly higher proportions of Hispanic respondents endorsed “some to very strong influence” than non-Hispanic White respondents need for more CM training (*p* = .023). Similarly, a significantly larger proportion of Hispanic respondents felt their agencies/administrations posed barriers to provision of CM (*p* = .005) relative to their non-Hispanic White counterparts. Differences by individual items on the CM-Supportive Statements subscale can be found in the Supplement.

## Discussion

Findings from our study revealed valuable insights into the variability of attitudes among Hispanic and non-Hispanic White providers towards CM as an evidence-based intervention for SUD. Although there has been limited CM research intentionally focusing on equity-related factors (e.g., ethnicity) at the provider-level, our findings align with prior work in other areas that have signaled the potential importance of providers’ ethnicity on beliefs and attitudes [21–23], which is key to address factors that may facilitate or impede implementation and the design of dissemination and implementation strategies.

Broadly, understanding providers’ beliefs and attitudes can be linked to the successful adoption and uptake of an intervention. This also includes identifying whether there are gaps or differences among providers, so that implementation strategies are tailored appropriately. Previous studies have cited that failure to adequately understand providers’ attitudes can hinder the successful implementation of an intervention [12]. In the context of present study several items on the CMBQ describe barriers that could be influenced by ones’ cultural background beliefs about CM evident by items such as “I find CM distasteful because it is basically paying someone to do what they should do already.” While other items (e.g., Our clinic rules prevent urine screening), may be more directly related organizational resources which could be influenced by the community characteristics (e.g., neighborhood socioeconomic status). Nevertheless, our findings, along with limited prior work [20], collectively and potentially demonstrate that it is not enough to simply tailor implementation strategies to fit within the practice context. Our results potentially support the need to tailor implementation strategies that consider the ethnicity or cultural background of SUD providers. Because few studies have examined equity-related factors beyond the client level, further research is needed to determine how to improve uptake and use of evidenced-based interventions among ethnoracially diverse providers.

The field of implementation science has recently called for equity-focused practices in the design and roll-out of strategies [26, 27]. Prior research has highlighted steps to the tailoring of implementation strategies, these include the assessment of determinants at the organizational level which influences practice or at the provider-level [28, 29]. However, there has been limited direction on how implementation strategies can be tailored to address equity-related factors at the provider-level. A possible solution to moderating negative attitudes towards CM, could be the integration of evidence which is often presented in trainings. Rather than simply sharing data on the effectiveness of CM, other forms of communication could be used to shift beliefs. For instance, storytelling which captures client and provider experiences with CM in ethnoracially-specific settings could also be incorporated and widely disseminated among providers. Another strategy may be the use of community participatory approaches that include providers as stakeholders to align CM practices with cultural values, which has been shown to be useful in Tribal communities [6].

There are several methodological limitations to the current study that should be noted and addressed in future studies. Among those being, the use of a convenience sample of SUD providers which lacked representativeness geographically and ethnoracially. For instance, we did not specifically include a question enquiring about where respondents were located and there was a significant amount of missing data (47%) in response to the item on organization name. For those that did respond, we were able to determine that the majority of respondents were located in the Pacific Northwest, U.S. Based on this, caution should be used when generalizing and interrupting findings on differences in beliefs. Further, the small number of respondents who identified as Black, American Indian/Alaska Native, Asian, and other ethnoracially diverse groups limited the study’s focus to only two groups, Hispanic and non-Hispanic white. As such, future research should examine differences in attitudes and beliefs about CM in a larger more diverse sample of SUD treatment providers. While some of the differences reported in this study could be attributed to working in various regions or in different practice settings, we controlled for agency type in our analyses, which is consistent with prior research. The “opt-in” design of the survey was not ideal and is not without risk of selection and response bias. Future studies could mitigate these concerns by widely disseminating surveys (e.g., geographically) to recruit a larger sample of participants and/or using a more targeted approach by engaging specific SUD agencies to participate.

## Conclusions

This study highlights the need for consideration of Hispanic ethnicity as a provider-level factor that may impact beliefs and attitudes towards CM, which has not been previously acknowledged in prior research focus on CM implementation. More largely, understanding the possible influence of providers’ ethnoraical background on the perceived advantages and disadvantages of CM is fundamental to developing equity focused implementation strategies that may aid the adoption of CM among SUD treatment providers.

### Electronic supplementary material

Below is the link to the electronic supplementary material.

Supplementary Material 1

## Data Availability

The data analyzed for current study is available from the corresponding author on reasonable request.
