# Supplementary material for: An unexplored equity factor: differential beliefs and attitudes toward contingency management by providers’ ethnicity
Source: BMC Health Serv Res. 2023 Aug 23;23:902. doi: 10.1186/s12913-023-09878-7 (PMC10464444; doi:10.1186/s12913-023-09878-7)
Supplement: Supplementary file 1 — Supplementary Material 1 [file 12913_2023_9878_MOESM1_ESM.docx]

Supplement Table 1. Proportions endorsing “Some to Very Strong Influence” per Contingency Management Beliefs Questionnaire (CMBQ) item within the CM-supportive Statements scale. P-values were derived from exact tests comparing responses from the non-Hispanic White and Hispanic groups.

|  | Non-Hispanic White | Hispanic | p-Value |
| --- | --- | --- | --- |
| CM-supportive Statements Scale |  |  |  |
| Any source of motivation, including extrinsic motivation, is good if it helps get clients involved and responding to treatment. | 96.5% | 98.3% | .647 |
| CM is useful when targeting treatment goals other than abstinence (attendance, activities). | 96.5% | 91.4% | .270 |
| CM is helpful because it helps keep clients engaged in treatment long enough for them to really learn valuable skills. | 94.0% | 96.6% | .700 |
| CM focuses on the good in clients' behavior, and not just what went wrong. | 91.7% | 96.6% | .310 |
| CM is good for clients because they get excited about their treatment and progress. | 92.9% | 96.5% | .475 |
| CM is worth the time and effort if it works. | 92.9% | 98.2% | .242 |
| I am in favor of adding CM interventions to our existing substance abuse treatment services. | 89.4% | 94.7% | .362 |
| CM helps clients get sober so that they can work on other aspects of treatment. | 89.3% | 96.6% | .200 |
| CM is useful when targeting abstinence. | 83.5% | 94.8% | .063 |
| CM will help get clients in the door (e.g., motivate them to come to treatment). | 90.6% | 98.3% | .083 |
| CM is good for the client-counselor relationship. | 87.1% | 94.7% | .160 |
